# Key mechanisms for chlamydia control in Guangdong, China: a mixed-methods causal-loop analysis

**DOI:** 10.1186/s12879-026-13471-8

**Published:** 2026-05-11

**Authors:** Yuting Wan, WenJun He, Peizhen Zhao, Tao Yang, Zizhen Huang, Xiaoshan Chen, Dadong Wu, Yunyun Xie, Jiatong Sun, Chunpin Li, Lexin Zhong, Zihan Lin, Xu Zhang, Xiaohui Zhang, Peibin Wu, Xian Huang, Jun Yuan, Woquan Chen, Chaofeng Nie, Dong Roman Xu, Shujie Huang, Cheng Wang

**Affiliations:** 1 School of Health Management, Southern Medical University, Guangzhou, China; 2 Dermatology Hospital, Southern Medical University, Guangzhou, China; 3 Acacia Lab for Implementation Science, School of Health Management, Southern Medical University, Guangzhou, China; 4 School of Public Health, the Key Laboratory of Environmental Pollution Monitoring and Disease Control, Ministry of Education, Guizhou Medical University, Guiyang, China; 5 Shenzhen Maternity and Child Healthcare Hospital, Southern Medical University, Shenzhen, China; 6 Acacia Lab for Implementation Science, School of Health Management and Dermatology Hospital, Southern Medical University, Guangzhou, China; 7 School of Public Health, Southern Medical University, Guangzhou, China; 8 The Fifth Affiliated Hospital, Southern Medical University, Guangzhou, China; 9 Public Health Medical Center of Puning, Puning, China; 10 The Third People’s Hospital of Zhuhai, Zhuhai, China; 11 Shenzhen Nanshan Center for Chronic Disease Control, Shenzhen, China; 12 Xinxing County Chronic Disease Prevention and Treatment Center, Xinxing, China; 13 Chronic Disease Prevention and Control Station of Xinyi City, Xinyi, China; 14 Southern Medical University Institute for Global Health, Guangzhou, China; 15 Center for World Health Organization Studies and Department of Health Management, School of Health Management, Southern Medical University, Guangzhou, China

**Keywords:** Multi-methodology approach, *Chlamydia trachomatis*, Causal loop diagram, Systems thinking, Structural analysis-MICMAC

## Abstract

**Background:**

The incidence of *Chlamydia trachomatis* (*C. trachomatis*) infections is increasing globally, with approximately 131 million new cases annually, leading to significant reproductive health complications. Evidence on the impact of chlamydia prevention and treatment programs in developing countries is scarce, leaving a critical gap in implementation. This study, conducted in Guangdong Province, China, aims to identify leverage points to facilitate evidence implementation by visualizing key factors and their interrelationships influencing chlamydia control through a causal loop diagram (CLD).

**Methods:**

In this mixed-methods study, variables were systematically identified through literature review (*n* = 15 studies), forming an initial conceptual model. Qualitative interviews with 70 stakeholders across five cities revealed implementation barriers and facilitators, refining the model. Second, a semi-quantitative MICMAC (Impact Matrix Cross-reference Multiplication Applied to Classification) analysis was employed to calculate link density and ranked influence-dependence measures.

**Results:**

The initial CLD consists of a main framework and four subsystems—Center for Public Health (CPH), Hospital, Target Audience, and the Government—comprising a total of 47 variables. A thematic analysis of the interview data revealed four major themes: health education, policy support, accurate identification of high-risk populations, and adherence. Two central feedback loops capture the core mechanisms. The first is a balancing loop involving policy requirements, community mobilization, and health education coverage and influencing variables such as condom promotion, infection rates, and disease burden. The second is a reinforcing loop driven by the public’s awareness of chlamydia prevention and control, amplifying the effects of educational outreach and stigma reduction efforts. Triangulation revealed alignment in health education centrality.

**Conclusions:**

These findings inform the development of context-specific interventions for chlamydia control in resource-limited settings. Health education, supported by policy and community engagement, may represent a central intervention. Targeted actions, such as expanding premarital and prenatal consultations and youth outreach in remote areas, could help reduce transmission through addressing stigma and other social barriers to care. Future system dynamics modeling is recommended to evaluate the long-term economic impact of chlamydia control and guide sustainable policy investment.

**Clinical trial number:**

Not applicable.

**Supplementary Information:**

The online version contains supplementary material available at 10.1186/s12879-026-13471-8.

## Background

*Chlamydia trachomatis* (*C. trachomatis*) is a common curable sexually transmitted infections (STI) and a significant cause of infertility, particularly in women, where it contributes to substantial tubal factor infertility [1]. Globally, chlamydia infections are increasing, with the WHO reporting approximately 131 million new cases annually [2]. In China, chlamydia represents a significant reproductive health burden, with 50,874 new cases reported in 2019–a 70.32% increase from 2008–corresponding to 55.32 cases per 100,000 people [3]. This notable prevalence highlights the need for effective prevention and control strategies.

Developed countries have implemented various strategies for chlamydia prevention and treatment, with evidence demonstrating their effectiveness. Since the 1990s, Sweden and the U.S. have established screening and early treatment as standard practices, reducing morbidity and costs [4, 5]. In the United Kingdom, the eSexual Health Clinic, which is based on a thorough understanding of chlamydia transmission, integrates partner notification, health promotion, and surveillance, leading to a 27% increase in total testing volume and a median time to treatment of only 1 day for 97% of managed patients [6]. Consistent with National Chlamydia Screening Programme outcomes, 2024 monitoring data show a 13.0% decrease in diagnoses versus 2023, with positivity among 15–24-year-olds falling from 9.6% to 8.8% [7].

However, in developing countries such as China, our understanding of the health strategies and mechanisms underlying chlamydia prevention and treatment remains limited [8, 9]. This is particularly true concerning the barriers to implementation and socioeconomic factors that may hinder the effectiveness of these strategies [10]. Although the “Pilot Program for the Prevention and Control of *C. trachomatis* in Guangdong Province (2022–2025),” launched by the Provincial Health Commission in Guangdong, China, included enhanced screening, standardized case management, provider capacity building, and implementation monitoring, evidence from complexity science and systems-based analyses suggests intervention effects are highly context-dependent and not readily generalizable across settings [11–13]. This knowledge gap is significant, as it impedes the development of tailored interventions that address the unique challenges faced in diverse settings. Chlamydia infections arise from dynamic interactions among interconnected variables, necessitating a systematic approach to map causal relationships in prevention and control [14]. By elucidating these relationships, we can identify leverage points for intervention, ultimately improving program effectiveness. Systems thinking tools, such as the causal loop diagram (CLD), provide a structured way to visualize interactions and feedback loops. Their application in addressing multifaceted public health challenges has been well documented [15]. For example, CLDs have been applied to examine systemic barriers to family planning utilization in Uganda [16].

The objective of this study was to explore the key factors influencing chlamydia control and their interrelationships through a causal loop diagram approach. Specifically, we aim to identify leverage points of comprehensive prevention strategies that can lead to effective control. By doing so, this study provides insights for enhancing chlamydia control efforts and informing international strategies across diverse health systems.

## Methods

The CLD represents system feedback structures, highlighting key variables, feedback loops, and leverage points [17]. Causal links were labeled as “+” (variables change in the same direction, ceteris paribus), “−” (change in opposite directions), and “//” (delayed effects). If a causal relationship loops back to affect the originating variable, it forms a closed sequence known as a feedback loop, which can be reinforcing (indicated by “R” for consistent effects) or balancing (indicated by “B” for counteracting effects), amplifying or stabilizing changes in the system; e.g., a R loop can increase screening uptake as awareness rises [18].

**Fig. 1:**
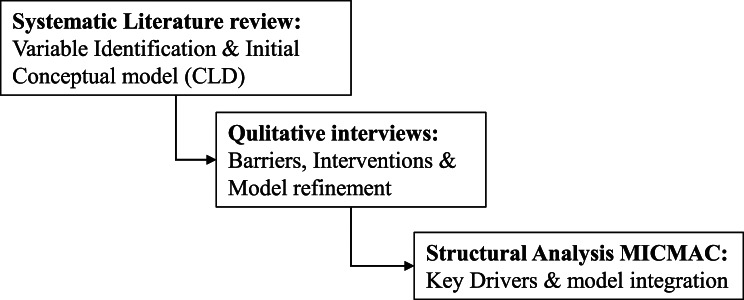
Framework of the multimethod system approach development

This study adopted an exploratory sequential mixed-methods design to construct a CLD. We adhered to the guidelines for good reporting of a mixed-methods study (GRAMM). First, a systematic literature review identifies factors and their causal links, allowing for the generation of a comprehensive CLD and assisting with interview outline generation. Second, qualitative interviews reveal barriers and facilitators, which helps refine the variables used for MICMAC data collection. Third, MICMAC served as the semi-quantitative component of the analysis, generating numerical outputs such as link density, agreement statistics, and ranked driver and dependence scores, which validated and structured the qualitative findings. These metrics informed the structural hierarchy and visual representation of the final CLD, integrating causal links, their relative strengths, and variable categories derived from the impact-dependence map [19]. Fig. 1 shows the framework developed. These steps are discussed in greater detail in the following sections.

### Initial conceptual model: causal loop diagram

#### Literature review

**Fig. 2:**
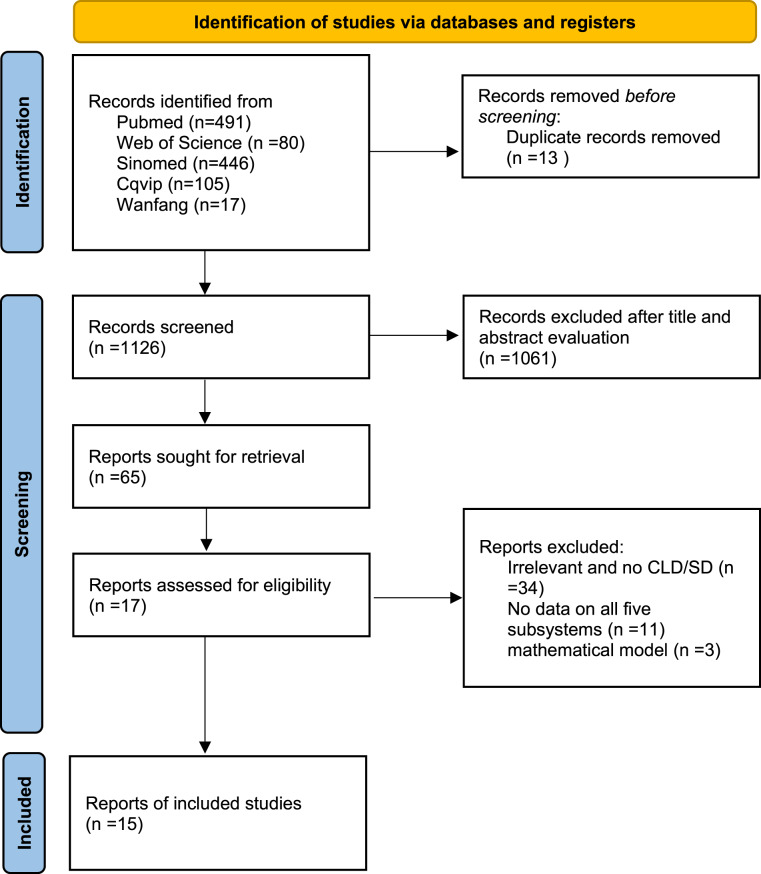
PRISMA flow diagram

We searched PubMed, Web of Science, CNKI, SinoMed, CQVIP, and Wanfang from inception to September 2022. The PRISMA flow diagram summarizes study selection (Fig. 2). The final search strategy and search records are available in supplementary materials 1. A literature extraction table was created on the basis of the four subsystems (see Supplementary Material 2).

#### Online group model building

Experts were purposively recruited for the online CLD-building meeting based on expertise in system dynamics, epidemiology, or chlamydia clinical management, and all provided informed consent before the session. In this study, primary variables were identified on the basis of individual or limited interdependencies reported in the literature. Each causal chain was reported by the extractors, connected by the modeling expert, and confirmed by the dermatologist. After the meeting, a draft was created via Vensim PLE (Version 6.3) software [20].

### Semi-structured interviews with providers

Between February and April 2023, we conducted semi-structured interviews using purposive sampling aligned with the four subsystems identified in the literature review. We recruited 70 stakeholders across five cities in Guangdong selected to capture variation in economic development and service contexts. Eligible participants were stakeholders involved in local chlamydia prevention and control activities (policy/administration, service delivery/management, and clinical care) and service users with relevant care experience.

The interview guide was developed and refined through online discussions with an expert panel, including a senior qualitative research PhD advisor, a dermatologist involved in the chlamydia pilot project and a baseline survey. On the basis of the study’s objectives, we used a deconstructed version of the initial CLD at the end of the interviews to gather feedback according to stakeholder attributes of the deconstructed CLD. Key domains addressed in the guide included stakeholder knowledge, scheme characteristics, internal and external environmental contexts, implementation processes, and feedback on the deconstructed CLD. For the details of the interview process, see supplementary Material 3.

We employed a thematic analysis approach using a top-down deductive method to achieve the study’s objectives, as the systematic literature review had already defined the boundaries and corresponding variables for the CLD [21]. Thematic analysis followed five steps: (a) iterative reading of transcripts to contextualize participants’ perspectives; (b) extraction of CLD-modifying statements; (c) thematic refinement and expansion based on participant feedback to the initial CLD; (d) theme nomenclature (without frequency thresholds); and (e) thematic mapping to the research framework (see Supplementary Material 4). For MICMAC preparation, candidate variables were screened using a pragmatic retention rule: variables mentioned at least three times across the interview dataset were advanced to matrix scoring. Because frequency does not necessarily indicate importance in qualitative analysis [22], lower-frequency variables were re-reviewed against the literature-derived CLD and retained in the narrative interpretation where needed to preserve plausible feedback pathways; only recurrent variables were advanced to MICMAC scoring [23].

### Structural analysis

The impact matrix cross-reference multiplication applied to a classification (MICMAC) method assesses the influence‒dependency relationships, categorizing variables into four types: influential (leverage points), relay, dependent, and autonomous (which can be excluded) [24]. Direct Influence ($D{I_i}$) and Direct Dependence ($D{D_i}$) were computed from the Matrix of Direct Influences (*M*) as the row sum and column sum for each variable, respectively ($D{I_i} = \mathop \sum \limits_j {M_{ij}}$,$D{D_i} = \mathop \sum \limits_i {M_{ji}}$). Indirect Influence ($I{I_i}$) and Indirect Dependence ($I{D_i}$) were computed analogously from the indirect effects matrix generated by MICMAC via iterative matrix multiplication (${M^k}$, k ≥ 2) until the influence–dependence values stabilized, capturing multi-step pathways and feedback effects. Higher influence indicates a greater impact on other factors, whereas higher dependence reflects greater reliance on others.

We ranked key variables in chlamydia infection prevention and control by assessing their direct and indirect influence on and dependence within the system. The MICMAC analysis followed three steps [25]: (a) Identifying and describing the main variables. (b) An expert panel independently scored pairwise relationships on a 0–3 scale, discrepancies were resolved through discussion and median scoring, with consensus requiring agreement by at least two raters (≥66.7%). The three key informants were purposively selected for their frontline expertise in clinical chlamydia management and public health practice. (c) Inputting matrix data into the official cloud-based MICMAC platform developed by La Prospective to rank direct and indirect effects and generate influence diagrams for causal chain integration [26].

### CLD and MICMAC integration

The MICMAC results were incorporated into the final CLD visualization through a three-step mapping process: (a) Variables were categorized and color-coded based on their quadrant position in the Influence-Dependence map: yellow for influential variables, black for relay variables, and orange for dependent variables. (b) Font sizes were adjusted to reflect systemic dominance, with larger font sizes assigned to the top three variables in the influence ranking. (c) The visual weight of causal links was calibrated according to the Matrix of Direct Influences (MDI) scores. Relationships were depicted as thin green arrows for weak influences (score = 1), medium light-blue arrows for moderate influences (score = 2), and thick dark-blue arrows for strong influences (score = 3). This integration enhances the final CLD by incorporating the strength and polarity of connections, revealing the categorization of variables on the basis of the influence‒dependence diagram.

## Results

### Search results and initial CLD

A total of 1,139 records were identified from five databases searched from inception to September 2022, and 1,126 remained after deduplication. Following the screening of titles and abstracts, 65 articles were further screened for full-text review. Ultimately, 15 studies were eligible for inclusion in the systematic review (see Fig. 2).

**Fig. 3:**
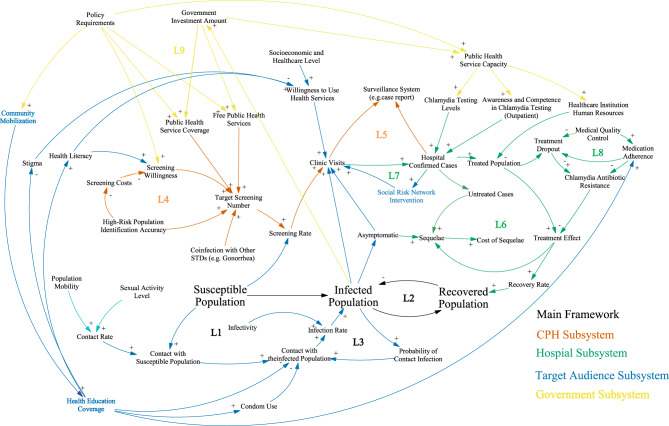
Initial CLD. “+” denotes a direct relationship, and “-” denotes a negative relationship

The initial CLD consists of a main framework and four subsystems—Center for Public Health (CPH), Hospital, Target Audience, and the Government—comprising a total of 47 variables (see Fig. 3).

The main framework (L1–L3) captures the core infection-recovery-susceptibility process via three feedback loops [27]; the CPH subsystem (L4–L5) models the operational trade-offs between screening volume, system capacity [28], and precision identification [29]; the hospital subsystem links diagnostic capability and partner notification (L7) to treatment adherence and the mitigation of antibiotic resistance (L6, L8) [30]; the target audience subsystem centers on behavioral drivers [31], where health education moderates contact rates by improving health literacy and reducing stigma [32]; and the government subsystem (L9) functions as an upstream regulator, using policy and investment to shape community mobilization and service capacity [33]. Detailed variable definitions and full causal chains are provided in Supplementary Material 2.

### Interview results

**Fig. 4:**
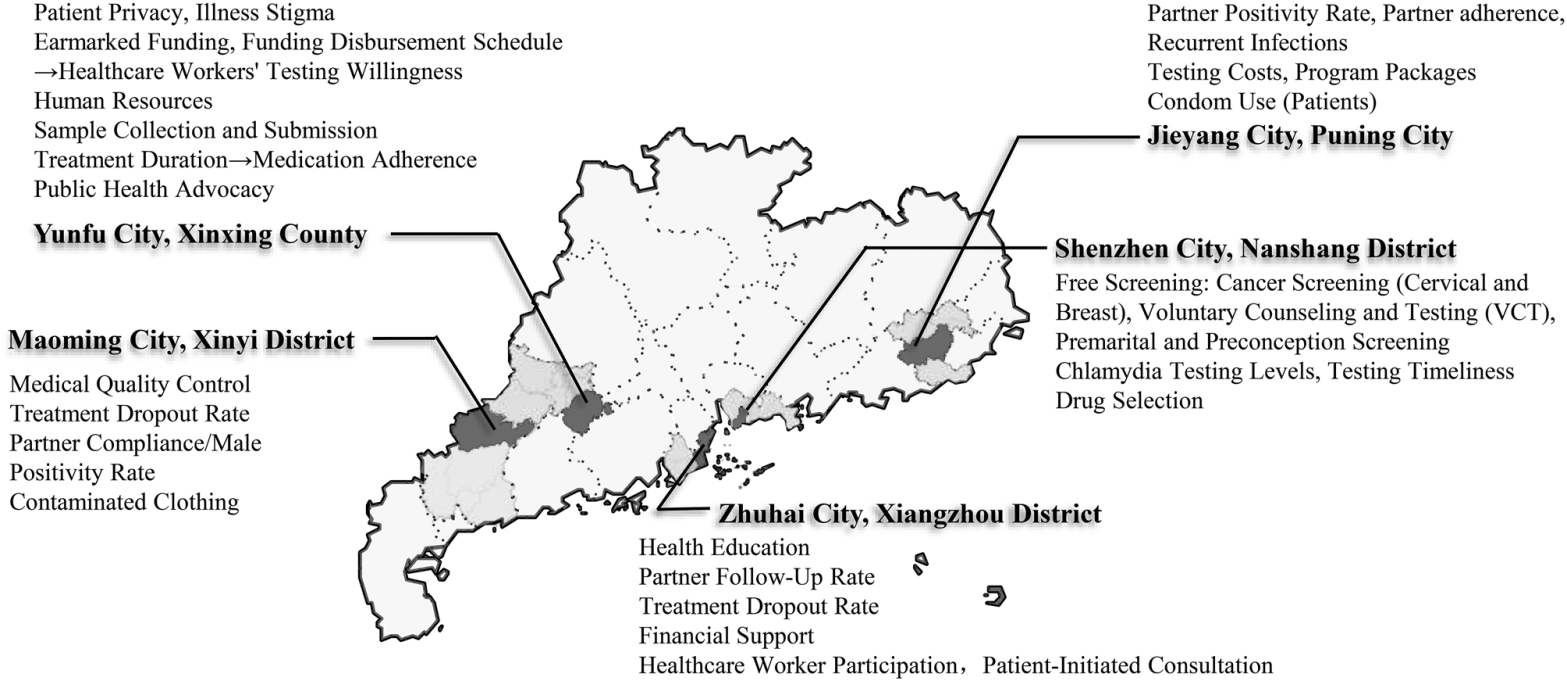
Initial impressions of the 5 cities interviewed. Map lines delineate study areas and do not necessarily depict accepted national boundaries

Seventy participants were recruited and interviewed (median age: 36 years; range: 22–58), with 14 participants from each region. The group included 10 outpatients (50% female), 5 nurses, 21 physicians, 4 other healthcare providers, 16 healthcare institution managers, and 14 health administrators (see Supplementary Material 5). Thematic saturation was achieved after approximately 60 interviews, with additional interviews conducted to ensure completeness. The interviewees were from five cities: Yunfu, Jieyang, Shenzhen, Zhuhai, and Maoming. A preliminary review of the interview materials from these locations involved recording keywords, which helped form an overall impression of the content (see Fig. 4).

By integrating word-cloud frequency analysis [34] with thematic coding, we identified 14 variables with a frequency of *n* > 2 (Supplementary Material 6). A further 12 factors were retained through qualitative synonym consolidation and preliminary cross-validation against the CLD (Supplementary Material 7), yielding a total of 26 core variables for inclusion in the MICMAC analysis.

**Fig. 5:**
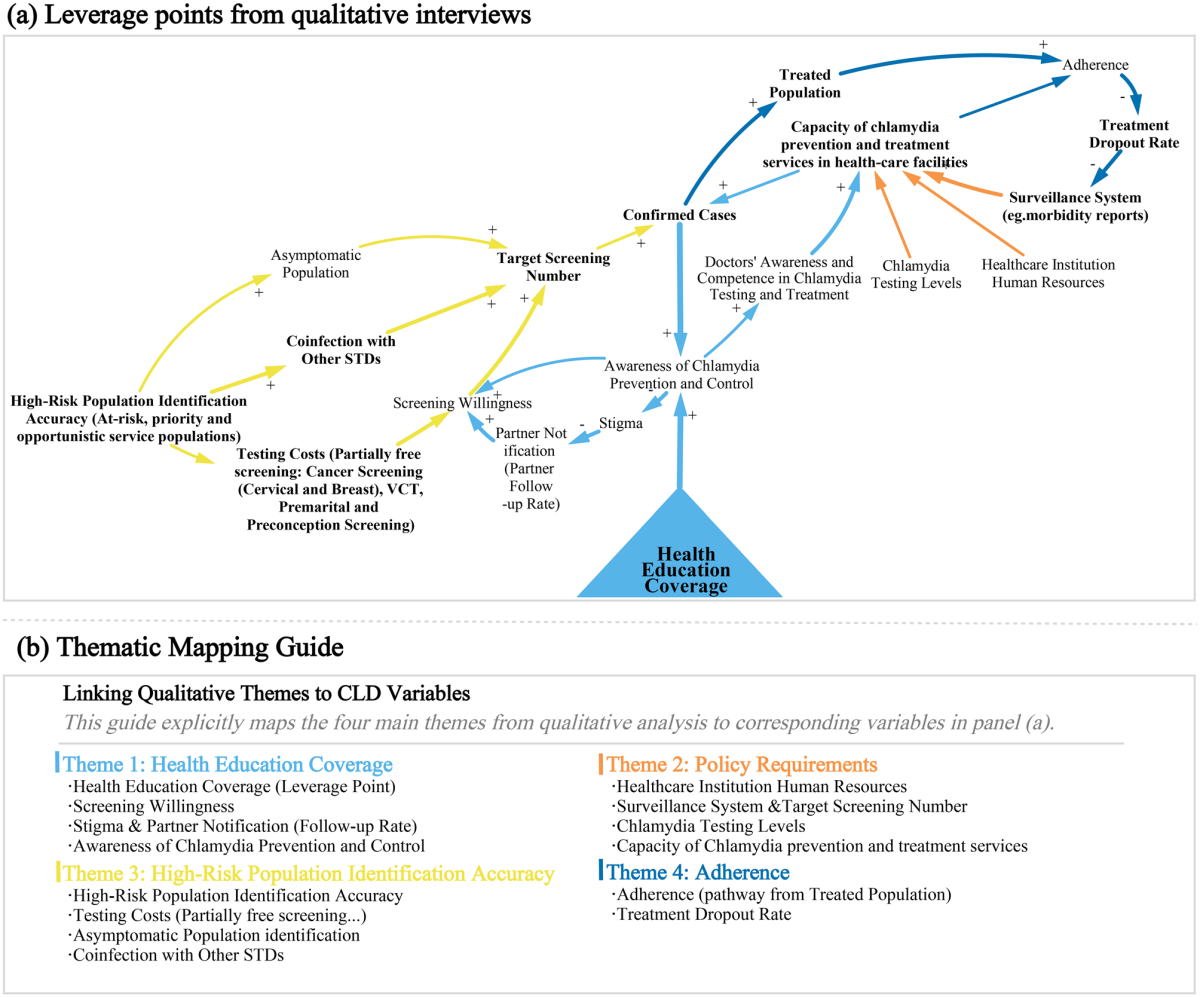
Multilevel determinants of chlamydia control synthesized from qualitative interviews. (**a**) Leverage points from qualitative interviews shaped as a balance scale, illustrating the dynamic interplay of system forces affecting chlamydia control. The blue triangle “Health Education Coverage” represents a key leverage point. The sign “+” denotes a direct relationship, and “-” denotes a negative relationship. (**b**) Thematic mapping guide providing an explicit linkage between the four main themes derived from qualitative analysis and specific key variables within the CLD structure shown in panel

Analysis of the interview transcripts identified and validated four main themes, as mapped to the CLD variables in Figure 5b: (1) health education, (2) policy support, (3) accurate identification of high-risk populations, and (4) adherence. Among these interventions, health education has emerged as the most critical intervention. The interview data were used to explain why the CLD variables might be associated with the chlamydia infection results. “The prevalence of health education” acts as a leverage point that drives the entire loop (see Figure 5a)

#### Health education coverage

Health Education Coverage increases Awareness of Chlamydia Prevention and Control, which in turn strengthens Screening Willingness, reduces Stigma, and facilitates Partner Notification (Partner Follow-up Rate). Participants highlighted both public outreach and in-clinic guidance as key delivery routes. They also emphasized provider training, noting that improvements in Doctors’ Awareness and Competence in Chlamydia Testing and Treatment can enhance chlamydia testing Levels and reinforce Confirmed Cases reporting and the Surveillance System (e.g., morbidity reports).

Stigma-related concerns were repeatedly mentioned as a barrier to Partner Notification (Partner Follow-up Rate): “We tell them that their partner definitely needs treatment. However, some people might think, ‘My husband or boyfriend does not have any symptoms, so if I tell him, will he think it is my fault?’ Therefore, they do not dare to mention it” (Physician, Yunfu). In Xinxing County (Yunfu), a predominantly rural county in western Guangdong Province, 6 of the 12 local staff participants reported stigma-related concerns as barriers to screening and partner notification. Respondents also described integrating screening and education into routine reproductive health services to improve reach and acceptability: “They want to have kids, right? Therefore, we conducted screenings as part of their premarital or pregnancy health checks. We also collaborate with the maternal and child health department to promote these policies” (Health administrator, Shenzhen).

#### Policy requirements

Interviewees emphasized that Policy Requirements and Government Investment need to be sustained beyond the pilot period to maintain momentum and enable scale-up. They noted that transitioning from a provincial pilot to broader implementation would require a clearer, locally adaptable implementation plan (e.g., differentiated screening strategies across districts) and stable financing arrangements. The provincial pilot programme document specifies that earmarked provincial funds are allocated annually during 2022–2025, with matching funds expected from participating localities and expenditures managed through dedicated accounts to ensure earmarked use. In interviews, administrators further indicated that decisions about continuation and scale-up are tied to interim performance evidence and formal health economic evaluation based on screening outputs. As one administrator put it, “One crucial task is to produce a health economic evaluation report … Only then can we secure continued investment and support for our project” (Health administrator, Shenzhen). Reported decision criteria commonly included routine feedback indicators such as screening positivity and detected infections, follow-up/enrolment among those testing positive, targets for standardized treatment, and monitoring of treatment outcomes.

#### High-risk population identification accuracy

Accurate identification of high-risk populations helps control screening costs and ensures efficient use of government funds, aligning with our systematic literature analysis. “Accurate targeting is essential. We want to ensure that limited resources are used where they are needed most. This is our main goal. Currently, the cost of the reagents we use is still quite high. With the COVID-19 pandemic, the public has become familiar with nucleic acid testing, but initially, they did not understand these concepts. Currently, they are more willing to undergo these highly sensitive screenings, but their cost is greater than that of antigen testing.

Experiences in expanding high-risk population screening include social mobilization, multidepartment cooperation, and bundled testing programs. “High-risk populations are already linked to basic public health services, maternal and child health, and key clinical departments, with testing primarily conducted in secondary or higher-level healthcare institutions. Screening for other populations depends on their willingness to participate, which requires social mobilization.” “For high-risk populations working in public venues, where intervention is challenging, we proactively coordinate with law enforcement to obtain relevant information and locate patients for treatment.” “Relying solely on our healthcare system, particularly dermatology and chronic disease institutions, may not be sufficient. We need specialized agencies at various levels to push government administrative departments to take action and secure their support.” “We’ve incorporated *C. trachomatis* testing into screening for men who have sex with men and into routine voluntary counseling and testing (VCT) packages. Nationwide, VCT primarily screens for HIV, but in Shenzhen, it is bundled with syphilis testing. In our clinic, we’ve also added nucleic acid testing for chlamydia and gonorrhea, making it a routine part of our screening program.”

#### Adherence

Views differed regarding adherence. Some respondents perceived treatment as straightforward, and believed that most patients follow recommendations: “The treatment is fairly simple, and … patients are usually compliant.” (Physician, Zhuhai). However, others emphasized barriers that undermine adherence in routine practice, particularly among asymptomatic individuals and during pregnancy: “Some asymptomatic patients are reluctant to accept treatment.” (Physician, Maoming) and “Some pregnant women refuse medication owing to concerns about side effects.” (Physician, Maoming). Beyond medication-taking, challenges were also described in partner management. One clinician noted that partner engagement may break down when partners are asymptomatic or test negative: “if the male partner tests negative [or is asymptomatic], he might refuse treatment,” (Physician, Jieyang).

To contextualize these perceptions, published evidence suggests that adherence gaps can be substantial. A prospective observational study reported that adherence to a 7-day doxycycline regimen was 77% among emergency department patients and 67% among female patients; medication adverse effects were among reported reasons for nonadherence, supporting interview concerns about side effects [35]. For partner-related follow-up, Clark et al. [33] found that 65% of participants notified their primary partner, yet after notification only approximately 33% of partners sought medical attention, consistent with our interview findings.

### MICMAC results

First, a systematic literature review and qualitative interviews were used to identify the variables applied in the MICMAC analysis. Table 1 lists the variable names, while their detailed operational definitions are provided in Supplementary Material 7.

**Table 1:** List of variables used in the MICMAC analysis

Variables (Long Label)	Variables (Short Label)
Policy Requirements	PolReq
Community Mobilization	ComMobil
Accuracy of High-Risk Population Identification	HRiskAcc
Coinfection with Other STI (VCT Testing, etc.)	CoInfect
Partner Notification (Partner Follow-up Rate)	PartNotif
Condom Promotion	CondPromo
Screening Rate	ScrRate
Health Education Coverage	HealthEdu
Chlamydia Prevention Knowledge Awareness Rate	ChlamKnow
Doctors’ Awareness of *C. trachomatis* testing and Standardized Diagnosis and Treatment Capability	DrChlam
Stigma	Stigma
Willingness to be Screened	ScrWilling
Compliance	Complian
Surveillance System	SurvSys
Chlamydi*a* testing Level	ChlamTest
Hospital Human Resources	HospHR
Chlamydia Resistance	ChlamRes
Disease Burden of chlamydia Infection	ChlamBur
Government Investment Amount	GovInvest
Testing Costs	TestCost
Population Mobility	PopMobil
Sexual Activity Level	SexAct
Contact Rate	ContRate
Infection Rate	InfRate
Confirmed Cases	ConfCases
Asymptomatic Population	AsymptPop

Second, an expert-scored 26 × 26 direct-influence matrix (676 cells; 650 possible off-diagonal links) was constructed (Supplementary Material 7). The consensus matrix had a link density of 52.2% (339/650 non-zero entries) and high inter-rater agreement (full agreement: 78.9%; ≥66.7% agreement: 100%), with disagreements resolved according to the consensus/median rule described in the Methods. Based on this matrix, we calculated the direct influence from immediate interactions (sum of row values), whereas indirect influence was derived through successive matrix multiplications (${M^k}$) to capture global feedback dynamics. The comparative rankings produced by the MICMAC software are shown in Table 2. As indicated in Table 2, “policy requirements” ranked first in both direct and indirect influence, “awareness of *C. trachomatis* testing and standardized treatment among doctors” ranked second in direct influence, and “coverage of health education” ranked second in indirect influence. These findings suggest that these three variables could serve as primary intervention strategies. The “level of *C. trachomatis* testing” and “hospital human resources,” which are difficult to change over time, are ranked at the bottom of the table. Additionally, “population mobility,” being primarily an external factor, has very low influence and dependency scores. The “burden of disease from chlamydia infection” ranks first in both direct and indirect dependence, while the “screening rate” ranks second in direct dependence, and “adherence” ranks second in indirect dependence. These findings indicate that these three variables may represent the main obstacles to intervention.

**Table 2:** Direct and indirect influences and dependency ratings according to the MICMAC method

Influence Rank				Dependence Rank			
	Direct		Indirect		Direct		Indirect
1	PolReq	1	PolReq	1	ChlamBur	1	ChlamBur
2	HealthEdu	2	HealthEdu	2	ScrRate	2	AsymptPop
3	DrChlam	3	ChlamKnow	3	ConfCases	3	ScrRate
4	ChlamKnow	4	ScrRate	4	AsymptPop	4	ConfCases
5	ScrRate	5	DrChlam	5	Colnfect	5	InfRate
6	Govlnvest	6	AsymptPop	6	InfRate	6	Colnfect
7	AsymptPop	7	Govlnvest	7	HealthEdu	7	Govlnvest
8	PartNotif	8	InfRate	8	ScrWilling	8	PartNotif
9	ComMobil	9	ChlamBur	9	Govlnvest	9	HealthEdu
10	Condpromo	10	ComMobil	10	PartNotif	10	DrChlam
11	InfRate	11	PartNotif	11	ChlamRes	11	CondPromo
12	Colnfect	12	CondPromo	12	CondPromo	12	ScrWilling
13	ChlamBur	13	Colnfect	13	DrChlam	13	PolReq
14	stigma	14	stigma	14	ChlamKnow	14	ChlamRes
15	HRiskAcc	15	HRiskAcc	15	PolReq	15	ChlamKnow
16	SurvSys	16	Scrwilling	16	ContRate	16	ContRate
17	Scrwilling	17	SurvSys	17	HRiskAcc	17	SurvSys
18	TestCost	18	ContRate	18	SurvSys	18	ComMobil
19	SexAct	19	ChlamRes	19	ComMobil	19	HRiskAcc
20	Complian	20	ConfCases	20	Complian	20	TestCost
21	ChlamRes	21	SexAct	21	TestCost	21	Complian
22	ContRate	22	TestCost	22	stigma	22	ChlamTest
23	ConfCases	23	Complian	23	ChlamTest	23	stigma
24	ChlamTest	24	ChlamTest	24	HospHR	24	HospHR
25	PopMobil	25	PopMobil	25	SexAct	25	SexAct
26	HospHR	26	HospHR	26	PopMobil	26	PopMobil

Direct Influence/Dependence were calculated as the row/column sums of the Matrix of Direct Influences (*M*), representing immediate relationships

Indirect Influence/Dependence were calculated from the indirect effects matrix produced by MICMAC via iterative matrix multiplication (${M^k}$, k ≥ 2) until stabilization, representing cumulative effects transmitted through multi-step pathways

Comparing direct and indirect rankings helps identify variables whose influence emerges primarily through indirect pathways

**Fig. 6:**
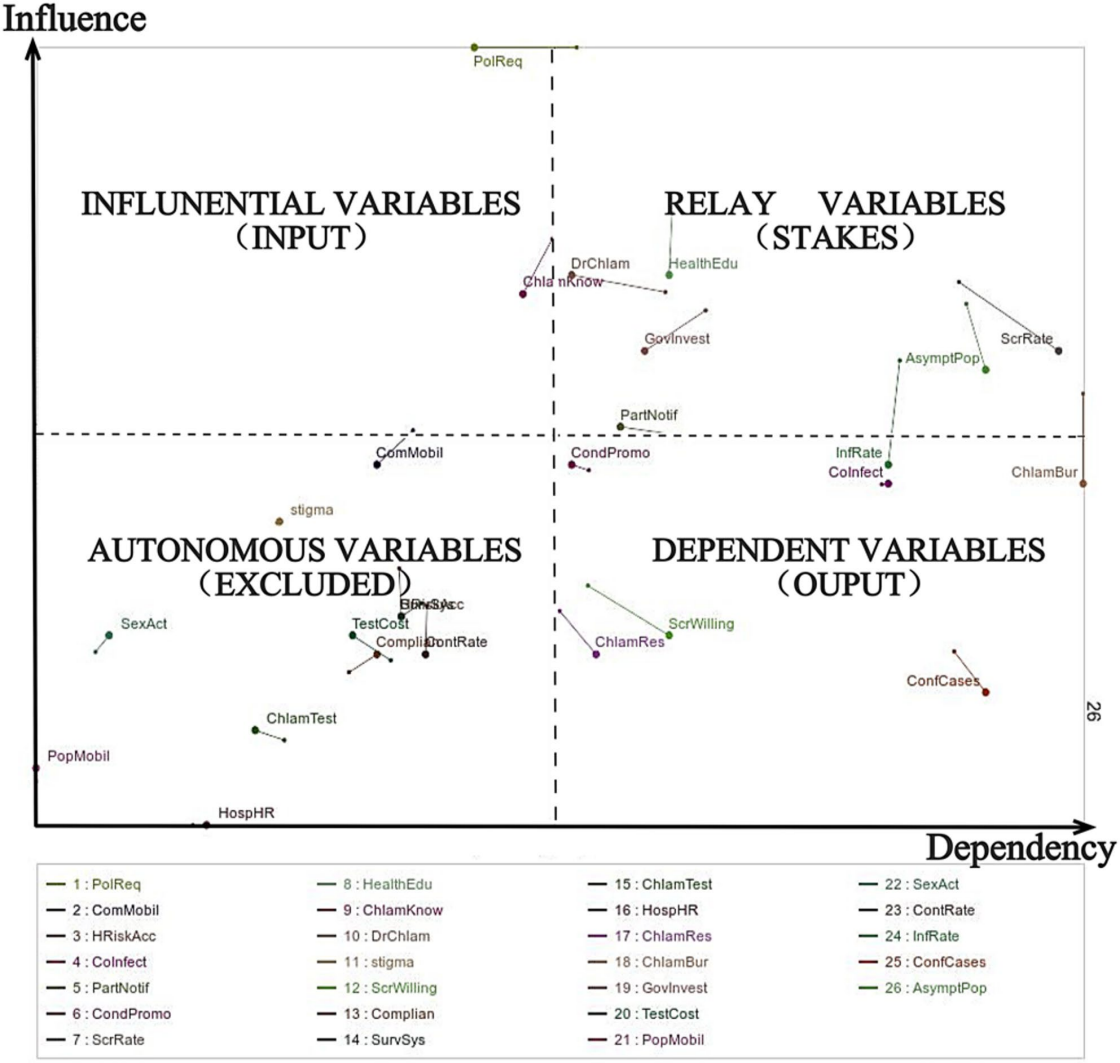
Direct-indirect influence-dependence diagram. a. The horizontal axis indicates dependencies, and the vertical axis indicates influences. b. Larger points indicate direct influence, and smaller points indicate indirect influence. ^c^. Quadrant 1 relay variables, quadrant 2 influential variables, quadrant 3 autonomous variables, quadrant 4 dependent variables

Finally, the variables were placed in a direct-indirect influence-dependence diagram (see Fig. 6) to visualize their overall influence-dependence ratings. The displacement diagram effectively combines direct and indirect influences, with larger dots representing direct influence and smaller dots indicating indirect influence. The horizontal axis represents dependence, whereas the vertical axis represents influence. On this basis, the 26 variables were divided into four quadrants: (1) Relay variables (stakes): Variables such as “Screening Rate,” “Asymptomatic Population,” and “Coverage of Health Education”—located in the first quadrant—are highly influential on the system while also being highly dependent on other variables. (2) Influential variables (input): Located in the second quadrant, these are the most critical variables. “Policy Requirements,” “Awareness of Chlamydia Knowledge,” and “Community Mobilization (indirect)” are variables that exhibit slight dependence but have a strong influence on other variables and the entire system when they change. (3) Autonomous variables (excluded): Autonomous variables located in the third quadrant are excluded because their overall influence and dependence are limited, and the closer they are to the lower left corner, the weaker their connection to other variables, such as “Population Mobility” and “Hospital Human Resources” (4). Dependent variables (output): Variables located in the fourth quadrant represent the system’s outputs, meaning that they are highly influenced by other variables and the system, such as “Diagnosed Cases,” “Screening Willingness,” and “Chlamydia Resistance.”

Of the 26 variables, 6 are relay variables, 3 are influential variables, 7 are dependent variables, and 10 are autonomous variables that were excluded (see Supplementary Material 8).

### Final CLD

**Fig. 7:**
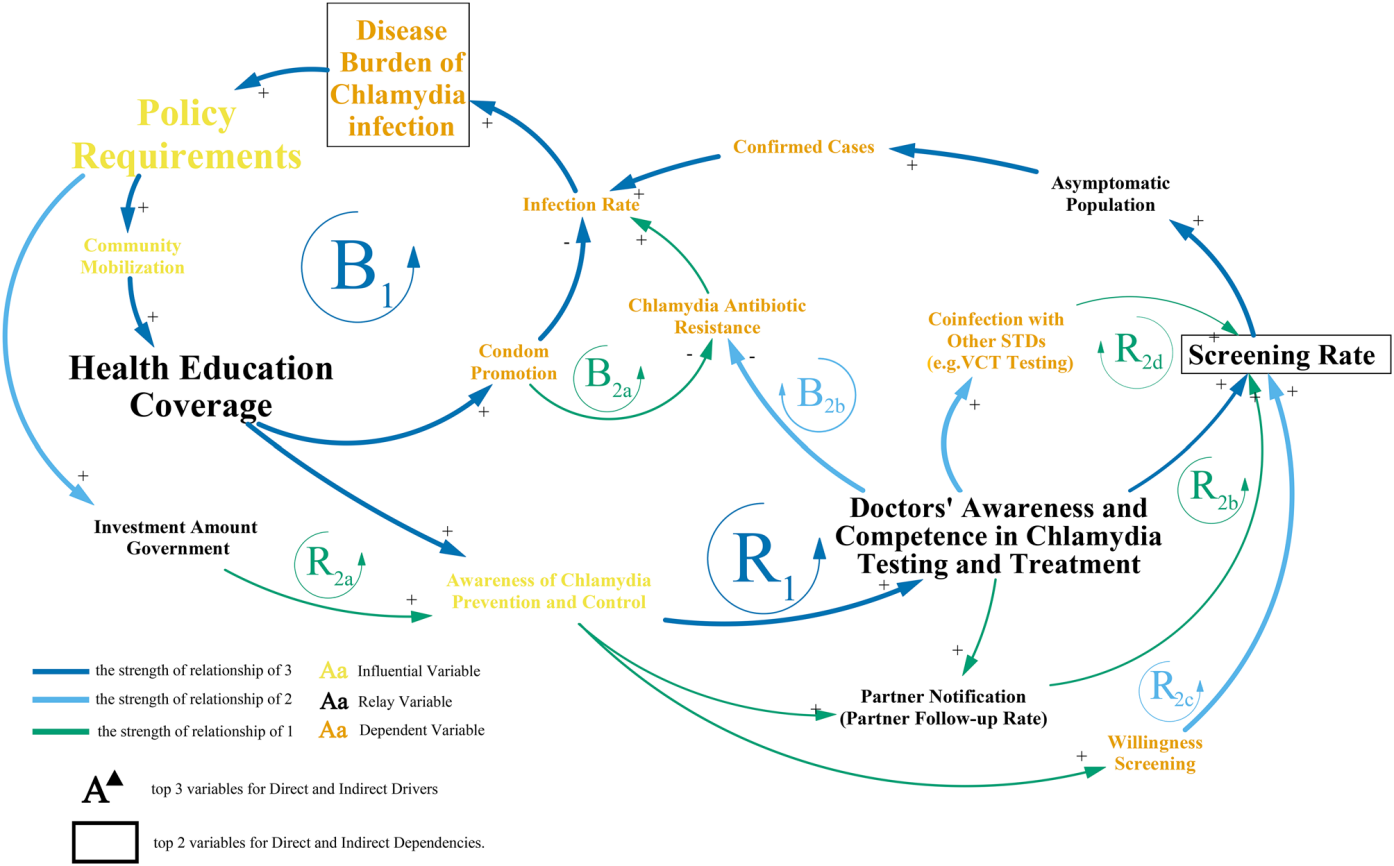
Final CLD. ^a^. “+” denotes a direct relationship, and “-” denotes a negative relationship. ^b^A feedback loop refers to a closed loop of causal pathways that can be traced back to the originating variable. “R” denotes reinforcing feedback that amplifies the direction of change, and ‘B’ denotes self-correcting balancing feedback. ^b^. Arrow colors indicate interaction strengths (3: dark blue; 2: light blue; 1: green) derived from matrix scores. Node colors signify the MICMAC structural classification (yellow: influential; Black: relay; orange: dependent). ^c^. Larger font is the top 3 variables for direct and indirect drivers, and larger boxes are the top 2 variables for direct and indirect dependencies

In the fourth step of the mixed-methods approach, a CLD was developed by integrating the findings from the MICMAC analysis into the initial CLDs, as depicted in Fig. 7. The final CLD identified two key feedback loops: Balancing Loop B1 (policy-driven infection reduction) and Reinforcing Loop R1 (awareness-driven screening enhancement), which were supported by five secondary strategies (see Fig. 7).

The first key feedback loop (B1) balances policy requirements, community mobilization, and health education coverage, with dependent factors including condom promotion, infection rates, and disease burden. This balancing feedback loop works to limit or correct changes within the system, ultimately aiming to reduce infection rates and alleviate the disease burden.

The second key feedback loop, R1, is a reinforcing loop. In this loop, the influencing factor is the awareness of chlamydia prevention and control knowledge. The relay factors include the awareness and adherence of doctors to *C. trachomatis* testing and standardized treatment, along with screening rates. The dependent factors are the asymptomatic population and confirmed cases. This reinforcing feedback loop amplifies the direction of change by increasing awareness of chlamydia prevention, thereby promoting early screening, diagnosis, and treatment.

The first strategy (R2) focuses on increasing screening rates. This strategy includes several components: increasing government investment (R2a), improving partner notification (R2b), increasing screening willingness (R2c), and implementing coordinated prevention and control measures for coinfections with other sexually transmitted diseases (R2d).

The second strategy targets *C. trachomatis* antibiotic resistance. The first approach (B2a) emphasizes prevention by promoting condom use, which may reduce recurrent infections and thereby lessen antibiotic resistance. The second approach (B2b) focuses on treatment, where standardized treatment by doctors and patient reminders may help reduce persistent infections, also contributing to a decrease in antibiotic resistance.

## Discussion

### Study objectives and significance

This study applies mixed methods to explore strategies for the prevention and treatment of chlamydia, with the goal of clarifying the complex relationships and influencing factors through the final CLD. The MICMAC analysis positioned disease burden as the primary downstream outcome (ranked 1st in direct dependence) and screening rate as the key relay node linking interventions to outcomes, supporting a strategy that prioritizes burden monitoring while targeting upstream levers. Systematic evidence identified health education coverage as the stakeholder leverage point, described by interviewees as the most critical intervention and ranked 2nd in direct influence.

### Interpretation of key findings

According to CLD (Fig. 7), we identified several key feedback loops. The first significant feedback loop (B1) is related to primary prevention. Health education coverage appears to be a central mechanism within this loop. Notably, the MICMAC matrix of direct influences (MDI) corroborates the structural strength of this loop, as all causal links within B1 were assigned the maximum influence rating of 3. This quantitative strength consistent with the qualitative mechanism: high-quality education improves awareness of chlamydia prevention, which in turn increases screening willingness and reduces stigma, thereby assisting partner management. The interviews emphasized the need to expand health education efforts, particularly through premarital and prenatal sessions, as well as outreach to younger populations in remote areas. High-quality, colloquial educational materials are essential for this effort [36]. Evidence from other settings suggests that sustaining preventive behaviors may require ongoing system-level support beyond short-term incentives [37], which is consistent with the central role of policy requirements within the B1 loop. The second key feedback loop (R1) focuses on secondary prevention, emphasizing early screening, diagnosis, and treatment. Improving chlamydia prevention and treatment knowledge among both patients and healthcare providers, particularly doctors’ awareness of testing and standardized treatment protocols, is crucial in this loop. Expanding asymptomatic testing requires multidepartment collaboration and integrated programs. Despite existing guidelines, implementation remains challenging. The interviewees proposed electronic treatment plans, reminders, training, incentives, and simplified testing.

In the implementation of chlamydia prevention and control strategies, it is imperative to consider the partner notification (R2b) relay variable in this feedback loop, as recurrent chlamydia infections after treatment are common, often due to reinfection from untreated partners in ongoing sexual relationships [38]. In fact, quantitative data indicate that men’s acceptance rates for testing may be lower than women’s acceptance rates, with notable differences: acceptance rates of 81.4% for women compared with only 29.4% for men [39]. Although patient privacy was not explicitly included in the causal loop diagram, it was identified as a bottleneck in all five locations during the qualitative interviews. A literature review indicated that effective chlamydia screening programs must provide clear, relevant information, present all testing options, and ensure anonymity [40]. These barriers may weaken the effectiveness of partner notification within the R2b loop, underscoring the importance of addressing gender differences and privacy concerns to sustain this feedback mechanism. Furthermore, stigma—often influenced by local cultural norms—appeared more salient in Xinxing County (Yunfu), a predominantly rural county in western Guangdong Province. In this site, stigma-related concerns were reported by 6 of the 12 local staff participants and were linked to reduced screening willingness and partner notification. Participants suggested that integrating privacy protection into routine reproductive health services may reduce anticipated stigma. More broadly, strengthening chlamydia prevention knowledge and awareness may help alleviate stigma-related barriers to screening and partner follow-up.

### Implications for practice

To address the disease burden, establishing a robust surveillance system to collect and analyze data on chlamydia incidence, prevalence, and risk factors is essential. As Marmot argued, these data can be utilized to inform evidence-based policy-making and resource allocation, enabling more targeted and effective public health interventions [41]. Furthermore, interventions targeting health education must extend beyond clinical settings. Schools play a vital role in reducing risky sexual behaviors and STI acquisition rates in adolescents [42]. Consequently, incorporating sexual health education, including information about chlamydia, into the school curriculum and encouraging adolescents to engage in extracurricular activities and awareness campaigns are important strategies to address this leverage point.

Feasibility warrants explicit consideration. Scale-up depends on locally aligned financing, administrative commitment, and credible performance signals. Implementation may be constrained by workforce shortages, uneven testing capacity, competing service priorities, and community-level barriers, which may reduce uptake in key populations [40].

### Limitations

One key limitation is that the causal loop diagram developed in this study is a qualitative model. Consequently, the loop polarity was not mathematically quantified, and the system’s behavior overtime remains untested via simulation. However, it provides valuable information on leverage points, which can inform future system dynamics modeling for chlamydia prevention and control. A significant challenge in this study was completing the MICMAC matrix. Initially, 47 variables were considered, requiring respondents to assess 2,162 interactions, which was nearly impossible to complete. This challenge was mitigated by using qualitative interviews to streamline the variable selection process. Potential selection bias is inherent in purposive sampling, which may limit representativeness. Although stakeholders were recruited across five cities with varying economic contexts, service users were underrepresented (10/70), potentially leading to insufficient capture of demand-side constraints. Similarly, the MICMAC analysis relied on a small panel, although prioritizing the deep implicit knowledge of these key informants, this may bias the identification and weighting of system relationships toward professionally salient pathways. Transferability beyond Guangdong should be assessed via validation in other provinces or comparable settings. Additionally, a limitation is that the qualitative interview data have not yet been coded by multiple researchers via the Consolidated Framework for Implementation Research (CFIR) coding framework, which will be addressed in future research.

## Conclusions

Key barriers to chlamydia control include low public awareness, stigma, insufficient healthcare resources, and fragmented stakeholder coordination, whereas enabling factors include health education, integrated services, and policy mandates. By synthesizing multisource data, this study develops a prevention model that advocates targeted health education, privacy protection, and systemic interventions. The integration of MICMAC analysis provided objective evidence identifying health education as the systemic leverage point for reducing the disease burden. This three-step CLD development approach provides a transparent framework for CLD construction, reducing subjectivity in variable selection and relationship mapping. The model supports stakeholders in visualizing and addressing complex challenges, specifically, by leveraging health education to enhance disease awareness, thereby mitigating stigma-driven barriers to partner notification. Ultimately, this study offers a transferable framework for optimizing STI control strategies in Guangdong and other settings with similar implementation contexts.

## Electronic supplementary material

Below is the link to the electronic supplementary material.

Supplementary material 1

Supplementary material 2

Supplementary material 3

Supplementary material 4

Supplementary material 5

Supplementary material 6

Supplementary material 7

Supplementary material 8

## Data Availability

Data is provided within the manuscript or supplementary information files.

## Abbreviations

*C.**trachomatis*: *Chlamydia trachomatis*
CLD: Causal loop diagrams
MICMAC: Matrix cross-reference multiplication applied to a classification
CPH: Center for Public Health
STI: Sexually transmitted infections
STDs: Sexually transmitted diseases
VCT: Voluntary counseling and testing
ANF: Audit and feedback
