# Supplementary material for: Key mechanisms for chlamydia control in Guangdong, China: a mixed-methods causal-loop analysis
Source: BMC Infect Dis. 2026 May 11;26:1247. doi: 10.1186/s12879-026-13471-8 (PMC13335349; doi:10.1186/s12879-026-13471-8)
Supplement: Supplementary file 1 — Supplementary material 1 [file 12879_2026_13471_MOESM1_ESM.docx]

**Search strategy and database search record**

**search terms**

The search terms are divided into two parts: the first part is "system dynamics" or "causal loop diagrams," limiting the focus to system dynamics or causal loop diagrams. The second part is "chlamydia" or "STDs," restricting the scope to the system dynamics or causal loop diagrams of chlamydia or sexually transmitted diseases.

**Criteria for selecting studies**

Inclusion Criteria: We included studies that explore factors related to the transmission, treatment, prevention, and policies of sexually transmitted diseases (STDs), particularly Chlamydia. Because the number of the literatures specific for Chlamydia is limited, we also include other STD papers, which will provide an overall picture of relative factors and deepen our understanding of Chlamydia’s prevention and treatment Studies that present their findings through causal loop diagrams, system dynamics, or systems thinking were preferred.

Exclusion Criteria: We excluded articles for which the full text was not available, those that had not undergone peer review (e.g., conference papers and preprints), studies focusing on diseases other than Chlamydia (e.g., blinding trachoma), or articles solely addressing the pathological mechanisms of Chlamydia.

**Database search record**

| **Search** | **PubMed Query 8-09-2022 _491** | **Items found** |
| --- | --- | --- |
| #5 | Filters: Chinese, English | 491 |
| #4 | #1 AND (#2 OR #3) | 498 |
| #3 | "sexually transmitted diseases/analysis"[MeSH Terms] OR "sexually transmitted diseases/complications"[MeSH Terms] OR "sexually transmitted diseases/economics"[MeSH Terms] OR "sexually transmitted diseases/epidemiology"[MeSH Terms] OR "sexually transmitted diseases/prevention and control"[MeSH Terms] OR "sexually transmitted diseases/statistics and numerical data"[MeSH Terms] OR "sexually transmitted disease*"[Title/Abstract] OR "venereal disease*"[Title/Abstract] OR "std"[Title/Abstract] OR "sti"[Title/Abstract] OR "sexually transmitted infection*"[Title/Abstract] | 219103 |
| #2 | "chlamydia/analysis"[MeSH Terms] OR "chlamydia/epidemiology"[MeSH Terms] OR "chlamydia/statistics and numerical data"[MeSH Terms] OR "chlamydia infections/analysis"[MeSH Terms] OR "chlamydia infections/complications"[MeSH Terms] OR "chlamydia infections/diagnosis"[MeSH Terms] OR "chlamydia infections/economics"[MeSH Terms] OR "chlamydia infections/epidemiology"[MeSH Terms] OR "chlamydia infections/statistics and numerical data"[MeSH Terms] OR "infection chlamydia"[Title/Abstract] OR "Chlamydia Infection"[Title/Abstract] | 166638 |
| #1 | "Systems Analysis"[MeSH Terms] OR "system dynamic*"[Title/Abstract] OR "agent based model*"[Title/Abstract] OR "agent based model*"[Title/Abstract] OR "causal loop diagrams"[Title/Abstract] | 105578 |

| **Search** | **Web of Science Query 3-09-2022 _80** | **Items found** |
| --- | --- | --- |
| #5 | #1 AND #4 and Obstetrics Gynecology or Infectious Diseases or Health Care Sciences Services or Public Environmental Occupational Health or Reproductive Biology or Management or Psychology Biological or Medical Informatics or Social Sciences Interdisciplinary or Behavioral Sciences or Health Policy Services or Medicine General Internal or Emergency Medicine | 80 |
| #4 | #1 AND (#2 OR #3) | 1826 |
| #3 | TS=("Sexually Transmitted Disease*") or TS=("Venereal Disease*") or TS=("STD*") or TS=("STI*") or TS=("sexually transmitted infection*") | 2312858 |
| #2 | TS= ("Chlamydia Infections") or TS= ("Chlamydia") or TS= ("infection chlamydia") or TS=("Chlamydia Infection") | 18931 |
| #1 | TS= ("Systems Analysis") or TS= ("System Dynamic*") or TS=("Agent-Based Modeling*") or TS=("agent based modeling*") or TS=("causal loop diagrams" ) | 22915 |

| **Search** | **sinomed 9-09-2022 _798** | **Items found** |
| --- | --- | --- |
| #5 | \| (("Chlamydia" OR "Sexually Transmitted Diseases" OR "Sexually Transmitted Infections" OR "STDs" OR "STIs" OR "Venereal Diseases" OR "Infectious Diseases") AND ("System Dynamics" OR "Causal Loop Diagram" OR "Systems Thinking" OR "Systems Analysis" OR "Agent-Based Modeling" OR "Complexity Analysis" OR "System Dynamics Modelling" OR "Systems Approach" OR "Systems Medicine" OR "Systems-Based Approach"))AND("R1" OR "R-" OR "R75" OR "R71") \| \| --- \| | 446 |
| #4 | #1 AND #2 | 798 |
| #2 | ("Chlamydia" OR "Sexually Transmitted Diseases" OR "Sexually Transmitted Infections" OR "STDs" OR "STIs" OR "Venereal Diseases" OR "Infectious Diseases") | 947815 |
| #1 | ("System Dynamics" OR "Causal Loop Diagram" OR "Systems Thinking" OR "Systems Analysis" OR "Agent-Based Modeling" OR "Complexity Analysis" OR "System Dynamics Modelling" OR "Systems Approach" OR "Systems Medicine" OR "Systems-Based Approach") | 62882 |

| **Search** | **Cqvip 2-09-2022 _105** | **Items found** |
| --- | --- | --- |
| #3 | (M=("System Dynamics" + "Causal Loop Diagram") OR R=("System Dynamics" + "Causal Loop Diagram")) AND (M=("Systems Thinking" + "Systems Analysis" + "Agent-Based Modeling" + "Complexity Analysis" + "System Dynamics Modelling" + "Systems Approach" + "Systems Medicine" + "Systems-Based Approach") OR R=("Systems Thinking" + "Systems Analysis" + "Agent-Based Modeling" + "Complexity Analysis" + "System Dynamics Modelling" + "Systems Approach" + "Systems Medicine" + "Systems-Based Approach")) AND (M=("Chlamydia" + "Sexually Transmitted Diseases" + "Sexually Transmitted Infections" + "STDs" + "STIs" + "Venereal Diseases" + "Infectious Diseases") OR R=("Chlamydia" + "Sexually Transmitted Diseases" + "Sexually Transmitted Infections" + "STDs" + "STIs" + "Venereal Diseases" + "Infectious Diseases")) | 105 |
| #2 | (Title OR Keywords = "Sexually Transmitted Diseases") OR (Abstract = "Chlamydia" OR "Sexually Transmitted Diseases" OR "Sexually Transmitted Infections" OR "STDs" OR "STIs" OR "Venereal Diseases" OR "Infectious Diseases") | 409053 |
| #1 | (M=("System Dynamics" + "Causal Loop Diagram") OR R=("System Dynamics" + "Causal Loop Diagram")) AND (M=("Systems Thinking" + "Systems Analysis" + "Agent-Based Modeling" + "Complexity Analysis" + "System Dynamics Modelling" + "Systems Approach" + "Systems Medicine" + "Systems-Based Approach") OR R=("Systems Thinking" + "Systems Analysis" + "Agent-Based Modeling" + "Complexity Analysis" + "System Dynamics Modelling" + "Systems Approach" + "Systems Medicine" + "Systems-Based Approach")) | 30297 |

| **Search** | **Wanfang 2-09-2022 _17** | **Items found** |
| --- | --- | --- |
| #4 | #1 AND #2 | 17 |
| #2 | (Subject: ("Sexually Transmitted Diseases" OR "Chlamydia" OR "Sexually Transmitted Infections" OR "STDs" OR "STIs" OR "Venereal Diseases" OR "Infectious Diseases") OR Title/Keywords: ("Sexually Transmitted Diseases" OR "Chlamydia" OR "Sexually Transmitted Infections" OR "STDs" OR "STIs" OR "Venereal Diseases" OR "Infectious Diseases")) | 500481 |
| #1 | (Subject: ("System Dynamics" OR "Causal Loop Diagram") OR Title/Keywords: ("System Dynamics" OR "Causal Loop Diagram")) AND (Subject: ("Systems Analysis" OR "Systems Thinking" OR "Agent-Based Modeling" OR "Complexity Analysis" OR "System Dynamics Modelling" OR "Systems Approach" OR "Systems Medicine" OR "Systems-Based Approach")  OR Title/Keywords: ("Systems Analysis" OR "Systems Thinking" OR "Agent-Based Modeling" OR "Complexity Analysis" OR "System Dynamics Modelling" OR "Systems Approach" OR "Systems Medicine" OR "Systems-Based Approach")) | 1384 |
