# Supplementary material for: Key mechanisms for chlamydia control in Guangdong, China: a mixed-methods causal-loop analysis
Source: BMC Infect Dis. 2026 May 11;26:1247. doi: 10.1186/s12879-026-13471-8 (PMC13335349; doi:10.1186/s12879-026-13471-8)
Supplement: Supplementary file 3 — Supplementary material 3 [file 12879_2026_13471_MOESM3_ESM.docx]

**3.1 Guide to interview with experts**

**Participant Inclusion Criteria:**

a) Administrative staff, healthcare providers, and outpatients (regardless of test results) who participated in or implemented the pilot project.

b) Participants who were informed about the prevention and control plan through various channels (e.g., mobilization meetings, brochures).

c) Participants who agreed to participate in the study and signed informed consent forms

Exclusion Criteria:

a) Individuals were unable to complete the entire interview due to work or time constraints.

b) Heavily accented or unable to understand Mandarin.

c) Temporary staff.

Researchers invited potential participants through a WeChat group established by the project liaison, where the study's purpose was explained. The liaison collected available times from participants, and the interviewers then coordinated to schedule interviews via Tencent Meeting.

**interviewers**

18 interviewers were recruited and assigned roles as primary and secondary interviewers after training and mock interviews. Each interview lasted approximately 30 minutes. Before the interview, the interviewer explained the purpose, provided relevant materials, and obtained informed consent from the participants. After each interview, the primary interviewer uploaded the recorded session, and the secondary interviewer prepared an interview summary based on the recording and transcription within 24 hours. Interviews continued until data saturation was achieved, as confirmed by the researchers.

**Pre-interviews**

Pre-interviews were conducted with an administrator, a physician, and a general staff member to test the readability and comprehensibility of the interview guide, which was further revised accordingly. The interview guides for the three participant groups were largely similar (see supplementary material B2.1). Questions explored participants' backgrounds, opinions on the "3331" project, internal and external factors affecting project implementation, and key implementation steps.

**Outline of Interviews with Administrators of the Pilot Project on Prevention and Control of Chlamydia in the Reproductive Tract in Guangdong Province**

Prior to the start of the interview, the investigator will explain the purpose of the interview and provide the respondent with relevant materials and an informed consent form. If the respondent has any questions, they should be clarified prior to the interview. Interviews will only be conducted with the written or verbal consent of the respondent.

**I. Starting points (2 questions)**

Q1. Firstly, could you briefly describe your department, your position and the specific responsibilities you have undertaken in the Chlamydia Control Pilot Project?

E.g.1.Community Health Service Center: Community Mobilization, Health Education, Screening

E.g.2.Chronic Disease Prevention and Treatment Hospital/Dermatology Hospital: project management, organization and coordination, technical guidance, personnel training, quality control, clinical diagnosis and treatment

E.g.3.Health Administrative Department: administrative management, organization and coordination, funds

Q2.What do you know about the harms and prevalence of genital chlamydia infection?

1. **Scheme characteristics (1 question)**

Q3. What are your views on the chlamydia prevention and control pilot project plan, including screening and standardized treatment?

- Feasibility, effectiveness, complexity: duration, scope and number of steps of the pilot project

E.g.Content of the screening plan: **Screening scope** (high - risk groups, sexually transmitted disease clinics, sexual partners, non - sexually transmitted disease patients, community), **Sample types** (urine, vaginal/cervical swabs, urethral swabs), **Result accuracy rate** (sensitivity, specificity), and **Methods of result notification**

- Costs: Screening costs, required consumables/equipment, human resources
- Plan adaptability: Whether it can be modified, customized or improved (localized), and whether it can be tested or piloted on a small scale (with the condition of revocation if necessary)
- Relative advantages: It has more advantages compared with other similar solutions (such as better effectiveness), and the solution is formulated by a trustworthy team.
- Strength and quality of evidence: The plan is proven to be scientific based on practice guidelines, published literature, expert opinions or other sources.

1. **internal environment (1 question)**

Q4.What methods does your department adopt to promote the chlamydia prevention and control pilot project including screening and standardized treatment?（Which internal resources will affect the implementation of the plan?）

(The aspects that can be prompted are as follows; you can follow up by asking ‘How exactly does it have an impact?’ )

- Relevant goals/indicators (the extent to which the indicators of the new plan are clearly communicated, implemented, and feedback to employees)
- Plans/improvement measures to ensure the successful implementation of the chlamydia prevention and control pilot project
- Resources: Human resources (the number of dermatologists/venereologists, professional technical personnel related to chlamydia screening/treatment), venue space for chlamydia screening, materials and equipment, information systems for data storage/managing/reporting/analysis, funds, etc.
- Mechanisms: Tangible or intangible incentives or rewards (monetary rewards for achieving screening/standardized diagnosis and treatment goals, honorary titles such as screening/treatment champions or pacesetters), and corresponding penalties for relatively poor screening/treatment performance.
- Compatibility with existing work: The integration of chlamydia screening and standardized treatment with current clinical work
- Coordination and communication: The forms through which doctors receive information related to chlamydia screening/treatment (formal meetings, informal oral communication, online communication, etc.), and the forms of training (guidance by dedicated personnel, self - study with electronic resources, etc.)

1. **External environment (1 question)**

Q5.What external environmental factors do you think will affect the implementation of the chlamydia pilot project, including screening and standardized treatment? (Please give specific examples)

- Politics: financial support, existing policies, whether to establish a network of contacts with other institutions (such as academic contacts), the degree of contact, the main content of the contact, and whether the organization encourages the establishment of contacts or not.
- Economy: technical conditions (testing laboratories), other sources of funding (such as horizontal/vertical funding, donations), etc.
- Culture: social values, customs, traditional concepts (people's attitudes towards chlamydia infection)

1. **Personal characteristics (1 question)**

Q6.Which roles do you think have the most influence in the implementation of the chlamydia prevention and control pilot project, including screening and standardized treatment?

- Describe the characteristics of this type of role/group
- How did they get involved? By appointment or spontaneously?
- What are their roles? What kind of activities do they carry out?
- What specific help have they provided for the implementation of the pilot project?

1. **Implementation process (2 questions)**

Q7.From the perspective of the implementation process of the pilot project, which steps do you think are relatively crucial? Could you elaborate on them?

- ①Plan；②Mobilization；③Execution；④Feedback mechanisms/reflect and comment

1. **Final question (2 questions)**

Q8.At the end of this interview, could you summarize the main bottlenecks in the implementation of the chlamydia infection prevention and control pilot project, including screening and standardized treatment? And what are the measures to overcome these bottlenecks? (Please share specific implementation measures based on your experience.)

Q9.(Show the casual loop diagram to the interviewees.) As for the XXX you've just referred to, do you believe the indication of this causal relationship is right? And is there anything to be complemented?

The interview is over. Thank you for your support and cooperation!

**3.2 deconstructed CLD**

| 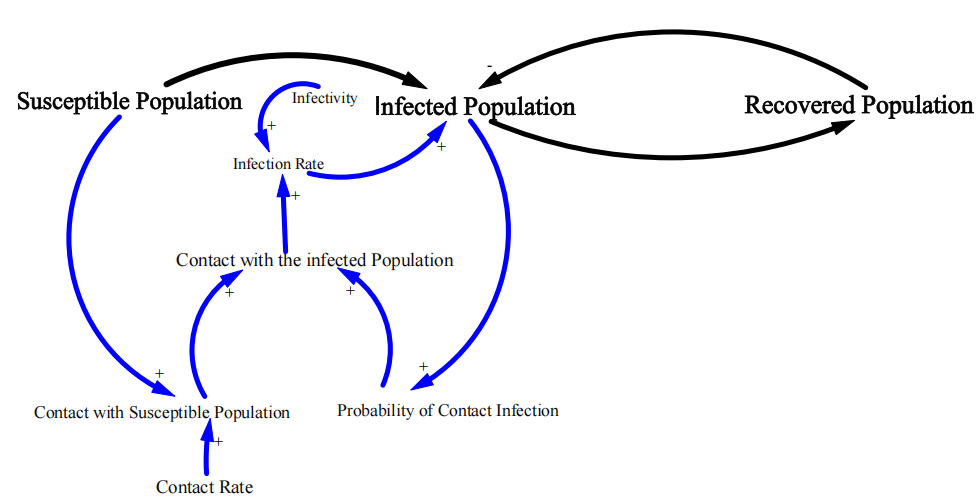 | 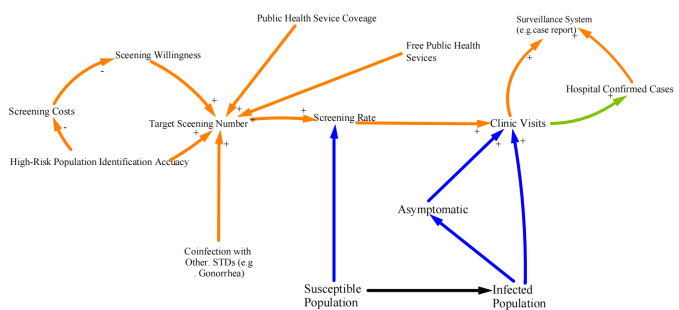 |
| --- | --- |
| 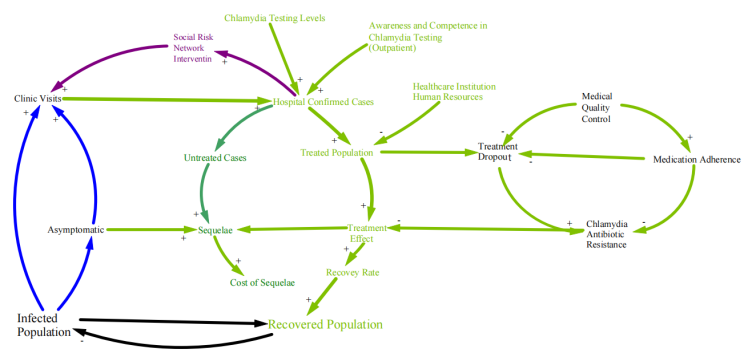 | 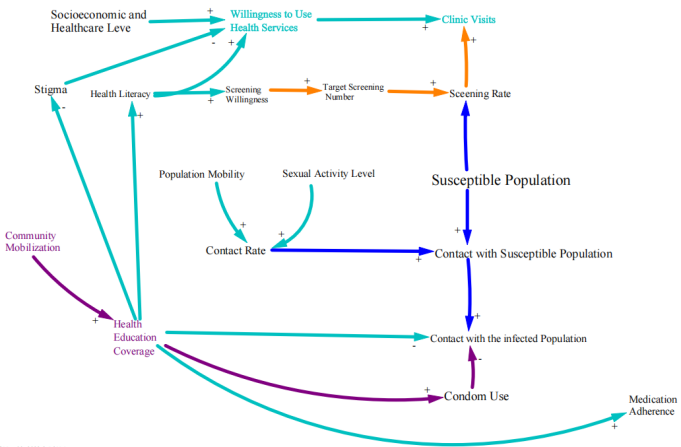 |
| 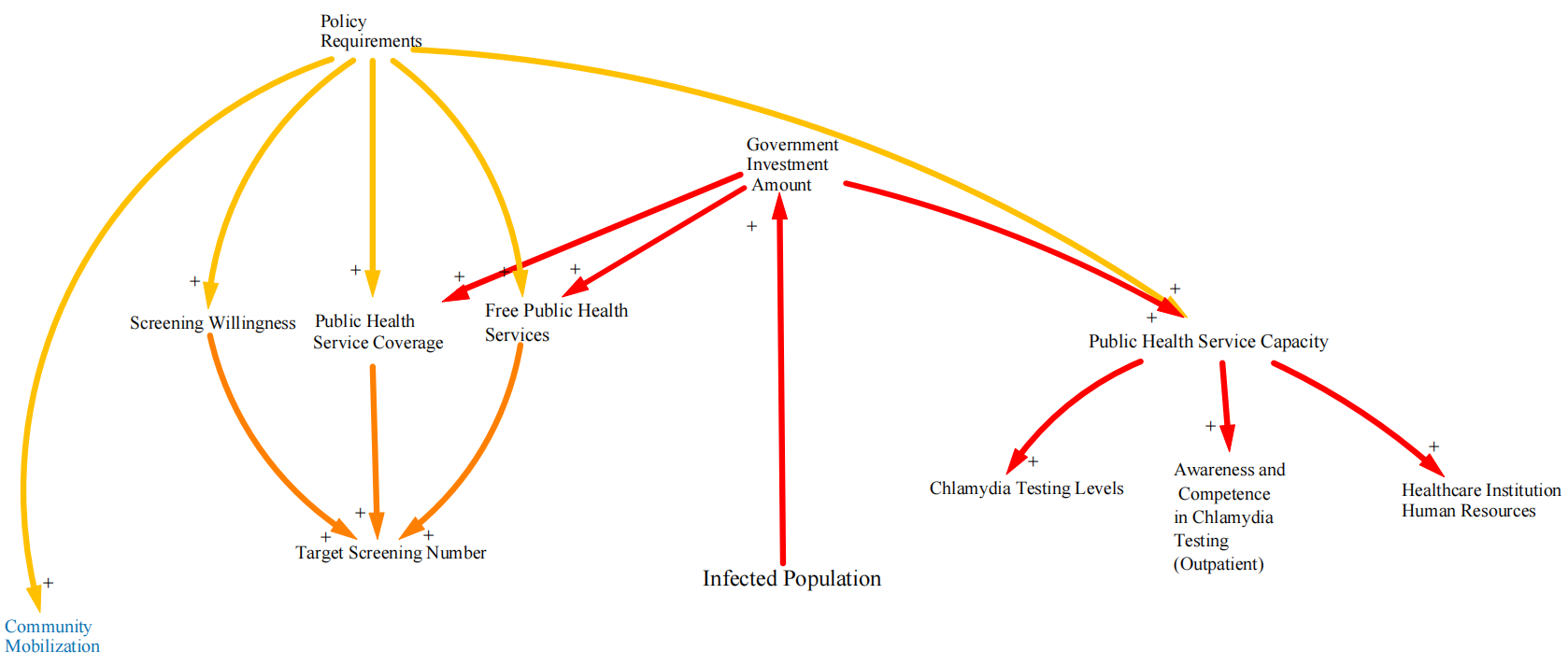 | |
