# Supplementary material for: Key mechanisms for chlamydia control in Guangdong, China: a mixed-methods causal-loop analysis
Source: BMC Infect Dis. 2026 May 11;26:1247. doi: 10.1186/s12879-026-13471-8 (PMC13335349; doi:10.1186/s12879-026-13471-8)
Supplement: Supplementary file 5 — Supplementary material 5 [file 12879_2026_13471_MOESM5_ESM.docx]

**Characteristics of Experts**

| **Region** | **Outpatient Patients** | **Healthcare Providers** | | |  | **Administrative Staff** | |  |
| --- | --- | --- | --- | --- | --- | --- | --- | --- |
|  |  | Nurses | Physicians | Others* |  | Healthcare Institution Managers** | Health administrators Personnel*** | Total |
| Yunfu City, Xinxing County | 2 | 0 | 5 | 1 |  | 4 | 2 | 14 |
| Shenzhen City, Nanshan District | 2 | 4 | 1 | 1 |  | 4 | 2 | 14 |
| Zhuhai City | 2 | 0 | 4 | 2 |  | 2 | 4 | 14 |
| Maoming City, Xinyi District | 2 | 0 | 6 | 0 |  | 5 | 1 | 14 |
| Jieyang City, Puning City | 2 | 1 | 5 | 0 |  | 1 | 5 | 14 |
| Total | 10 | 5 | 21 | 4 |  | 16 | 14 | 70 |

*** Others** include laboratory physicians and midwives. **** Healthcare Institution Managers:** Heads of departments such as AIDS Prevention, Dermatology and Venereology, Gynecology, Women's Health, Premarital and Preconception Health, Public Health, Health Care, Nursing, and Medical Affairs in local hospitals. ***** Health Administrative Personnel:** Employed in local Health and Family Planning Commissions and Centers for Disease Control and Prevention.
