# Supplementary material for: Key mechanisms for chlamydia control in Guangdong, China: a mixed-methods causal-loop analysis
Source: BMC Infect Dis. 2026 May 11;26:1247. doi: 10.1186/s12879-026-13471-8 (PMC13335349; doi:10.1186/s12879-026-13471-8)
Supplement: Supplementary file 6 — Supplementary material 6 [file 12879_2026_13471_MOESM6_ESM.docx]

**Word frequency**

| word | frequency |
| --- | --- |
| health education | 17 |
| screening costs/testing fees/free services | 10 |
| partner follow-up rates/partner notification | 6 |
| medication adherence | 6 |
| screening willingness | 6 |
| stigma | 6 |
| treatment dropout rates | 4 |
| healthcare worker awareness/proactive involvement | 4 |
| health literacy/awareness rates | 4 |
| human resources | 4 |
| Chlamydia testing levels | 4 |
| asymptomatic cases | 4 |
| financial support | 3 |
| antibiotic resistance | 3 |
| patient-initiated consultations | 2 |
| Awareness and Competence in Chlamydia Testing | 2 |
| Clinic Visits | 2 |
| Medical Quality Control | 2 |
| Chlamydia testing levels and efficiency | 2 |
| condom use | 2 |
| Standardized treatment of chlamydia STDs | 2 |
| Contact with Infected Population | 1 |
| High-Risk Population Identification Accuracy | 1 |
| Public Health Service Coverage | 1 |
| multiple sexual partners | 1 |
| Coinfection with Other STDs | 1 |
| sexual life | 1 |
