# Supplementary material for: Key mechanisms for chlamydia control in Guangdong, China: a mixed-methods causal-loop analysis
Source: BMC Infect Dis. 2026 May 11;26:1247. doi: 10.1186/s12879-026-13471-8 (PMC13335349; doi:10.1186/s12879-026-13471-8)
Supplement: Supplementary file 7 — Supplementary material 7 [file 12879_2026_13471_MOESM7_ESM.docx]

| **7.1 Detailed descriptions and operational definitions for all 47 variables included in the Causal Loop Diagram and MICMAC analysis.** | | |
| --- | --- | --- |
| **Variables (Long Label)** | **Variables (Short Label)** | **Variable Description** |
| Policy Requirements | PolReq | Policies related to the prevention and control of genital chlamydia infections. |
| Community Mobilization | ComMobil | Collaboration with organizations outside health services, such as partnering with the Women's Federation and township governments to enhance credibility, recruiting volunteers for advocacy and mobilization, and obtaining information on opportunistic service populations from public security authorities. |
| Accuracy of High-Risk Population Identification | HRiskAcc | Key populations: marital care group, prepregnancy care group, postabortion care group, prenatal care group, and infertility group indicators. High-risk populations: (1) Outpatients with urogenital infections: those in dermatology, gynecology, andrology, urology, or proctology clinics in pilot areas who meet one of the following conditions: ① symptoms suggestive of STI; ② coinfection with other STI; ③ sexual partner with STI; ④ high-risk sexual behaviors, such as multiple sexual partners or unprotected sex with new partners of unknown status. (2) Men who have sex with men: males aged 16 and above who have had insertive oral or anal sex with other men in the past year. (3) Female sex workers: females aged 16 and above who have provided sexual services to men in mid-to-low-end establishments such as foot massage parlors, leisure massage rooms, hair salons, and roadside shops for financial or other benefits in the past year. |
| Coinfection with Other STI (VCT Testing, etc.) | CoInfect | Integrate chlamydia infection prevention with reproductive health, maternal and child health care, and other STI prevention efforts for coordinated control. |
| Partner Notification (Partner Follow-up Rate) | PartNotif | Partner notification involves identifying the sexual contacts of STI patients, informing them, and encouraging them to undergo necessary screening, diagnosis, and treatment. If partners are not tested and treated, the risk of reinfection remains high even if the patient has been treated. |
| Condom Promotion | CondPromo | Vigorous promotion and widespread use of condoms is an effective measure for preventing and controlling sexually transmitted genital chlamydia infections. It is a low-cost, high-benefit intervention. |
| Screening Rate | ScrRate | Screening involves using quick and simple tests to identify suspected patients or those with defects from an ostensibly healthy population. Screening tests are not diagnostic but preliminary checks; positive or suspicious results require confirmatory testing and subsequent treatment. |
| Health Education Coverage | HealthEdu | Coverage of chlamydia-related health education, including education for susceptible populations, medical personnel, and administrative personnel. Main forms include: 1. Knowledge dissemination during premarital and prepregnancy care, cervical and breast cancer screening, and VCT testing (explaining its impact on reproductive health). 2. Publicity of beneficial policies. 3. Doctors educating diagnosed patients and their partners about chlamydia (explaining that it can be transmitted not only sexually but also through the eyes, respiratory tract, or even clothing, and that partners should be treated together to prevent reinfection). 4. Training for specialists and general practitioners. |
| Chlamydia Prevention Knowledge Awareness Rate | ChlamKnow | The proportion of patients and doctors who have a comprehensive understanding of the harm, transmission routes, prevention, and treatment measures of chlamydia. |
| Doctors' Awareness of *C. trachomatis* testing and Standardized Diagnosis and Treatment Capability | DrChlam | The ability of doctors in premarital and prepregnancy examination clinics, gynecology, obstetrics, urology, dermatology and venereology, andrology, and reproductive health clinics to recognize chlamydia infections based on clinical symptoms and medical history, their awareness of *C. trachomatis* testing, and the proportion of chlamydia-positive patients receiving standardized treatment (standardized treatment follows the "Clinical Diagnosis and Treatment Guidelines for Sexually Transmitted Diseases" issued by the Chinese Center for Disease Control and Prevention's STD Control Center). |
| Stigma | Stigma | Because one of the transmission routes of genital chlamydia infection is sexual transmission, a diagnosis might cause stigma, making some people unwilling to disclose it to others. The attitude toward chlamydia infection might differ between married and unmarried individuals. |
| Willingness to be Screened | ScrWilling | The willingness of three groups (high-risk populations, key populations, and opportunistic service populations), people coinfected with other STI, and the sexual partners of diagnosed patients to get tested for chlamydia. Note: This includes cases where individuals are unwilling to be screened due to the need to provide private information for the project. |
| Compliance | Complian | 1. Whether diagnosed patients follow medical advice to take medication for a 10-14 day course and avoid sexual activity for 28 days. 2. Whether the sexual partners of diagnosed patients are also taking medication. |
| Surveillance System | SurvSys | Establishing a surveillance system for the prevention and control of chlamydia infections. |
| Chlamydi*a* testing Level | ChlamTest | The ability to detect patients already infected with chlamydia. This includes both technical and organizational aspects. The technical aspects include the number of medical institutions conducting *C. trachomatis* testing (nucleic acid testing/antigen testing), the number of tests performed, and the positivity rate, the number of nucleic acid testing laboratories, and participation in chlamydia laboratory proficiency testing organized by relevant institutions (such as the national reference laboratory, provincial clinical testing centers, or disease control agencies). Organizational aspects include the rationality of sampling and submission procedures and the efficiency of test reporting. |
| Hospital Human Resources | HospHR | The number of medical personnel available to participate in chlamydia infection prevention and control. Shortages of doctors or heavy workloads may affect other aspects of chlamydia control efforts. |
| Chlamydia Resistance | ChlamRes | Drug tolerance during treatment. Currently, evidence for chlamydia resistance is insufficient, but chlamydia's resistance to treatment might involve mechanisms beyond resistance genes, possibly forming a shield-like state that makes it difficult for drugs to penetrate, similar to fungal spores. The resistance mechanisms are not fully understood and require further research. |
| Disease Burden of chlamydia Infection | ChlamBur | The epidemiological and economic burden of chlamydia infections. |
| Government Investment Amount | GovInvest | Government funding allocated based on baseline conditions and the effectiveness of prevention and control efforts in different regions. |
| Testing Costs | TestCost | The cost of *C. trachomatis* testing. First, testing costs vary by method, with nucleic acid testing being more expensive. Second, there are two situations: 1. Free testing for eligible individuals included in baseline surveys, and some premarital, prepregnancy, and cervical and breast cancer screenings. 2. Out-of-pocket costs for patients in combined STI testing packages and the sexual partners of chlamydia-positive patients. |
| Population Mobility | PopMobil | Migrant populations can be divided into in-migrants (nonresidents coming to the area) and out-migrants (residents leaving the area). Migrant populations can also be classified by duration: long-term (residing for an extended period, e.g., 5 years) and short-term migrants. Currently, the administrative management of migrant populations is primarily handled by public security departments, focusing on security issues. Other aspects, such as labor and social security, health, education, and family planning, are managed by respective departments. |
| Sexual Activity Level | SexAct | The number of sexual activities within a specific period. |
| Contact Rate | ContRate | The number of contacts per average infectious period. |
| Infection Rate | InfRate | The proportion of people currently infected with chlamydia within a tested population at a certain time, usually expressed as a percentage. In the susceptible - infectious - recovered model model, a susceptible individual has the potential to be infected upon contact with a patient. In modeling, it is assumed that each susceptible individual has an equal probability of contact with each patient at any time and a certain probability of transmission. The infection rate is the constant probability of contact and transmission per unit time. |
| Confirmed Cases | ConfCases | The number of individuals testing positive for chlamydia infection. |
| Asymptomatic Population | AsymptPop | Many patients infected with chlamydia are asymptomatic, which can affect various aspects of prevention and control efforts. |

**7.2 Cross-impact matrix of direct influences (MDI)—dark blue indicates a strong, light blue a weak**

1. The matrix shows the degree of influence of the “row variables” on the “column variables” rated from 0 (no influence), 1 (weak influence), 2 (moderate influence), 3 (strong influence).

influency

1. Influency Di=∑fi ; Dependency Rj=∑fj
