# Supplementary material for: Key mechanisms for chlamydia control in Guangdong, China: a mixed-methods causal-loop analysis
Source: BMC Infect Dis. 2026 May 11;26:1247. doi: 10.1186/s12879-026-13471-8 (PMC13335349; doi:10.1186/s12879-026-13471-8)
Supplement: Supplementary file 8 — Supplementary material 8 [file 12879_2026_13471_MOESM8_ESM.docx]

**Summary table of three-stage variables**

| **No.** | **Literature Variable** | **Interview Refining** | **MICMAC Quadrant** |
| --- | --- | --- | --- |
| 1 | Susceptible Population | Delete | — |
| 2 | Contact Rate | Retain | Autonomous Variable |
| 3 | Contact with Susceptible Population | Delete | — |
| 4 | Contact with the Infected Population | Delete | — |
| 5 | Infection Rate | Retain | Dependent Variable |
| 6 | Infected Population | Delete | — |
| 7 | Infectivity | Delete | — |
| 8 | Probability of Contact Infection | Delete | — |
| 9 | Recovered Population | Delete | — |
| 10 | Asymptomatic | Asymptomatic Population | Relay Variable |
| 11 | Sequelae | Delete | — |
| 12 | Cost of Sequelae | Disease Burden of Chlamydia Infection | Dependent Variable |
| 13 | Target Screening Number | Delete | — |
| 14 | Screening Rate | Retain | Relay Variable |
| 15 | Screening Willingness | Retain | Dependent Variable |
| 16 | Screening Costs | Testing Costs | Autonomous Variable |
| 17 | High-Risk Population Identification Accuracy | Retain* | Autonomous Variable |
| 18 | Coinfection with Other STDs  (e.g. Gonorrhea) | Coinfection with Other STDs  (e.g.VCT Testing) | Dependent Variable |
| 19 | Clinic Visits | Delete | — |
| 20 | Hospital Confirmed Cases | Confirmed Cases | Dependent Variable |
| 21 | Surveillance System | Retain | Autonomous Variable |
| 22 | Treated Population | Delete | — |
| 23 | Treatment Effect | Delete | — |
| 24 | Recovery Rate | Delete | — |
| 25 | Chlamydia Testing Levels | Retain | Autonomous Variable |
| 26 | Awareness and Competence in Chlamydia Testing (Outpatient) | Doctors' Awareness and Competence in Chlamydia Testing and Treatment | Relay Variable |
| 27 | Social Risk Network Intervention | Partner Notification  (Partner Follow-up Rate) | Relay Variable |
| 28 | Healthcare Institution Human Resources | Retain | Autonomous Variable |
| 29 | Untreated Cases | Delete | — |
| 30 | Medication Adherence | Adherence* | Autonomous Variable |
| 31 | Medical Quality Control | Delete | — |
| 32 | Treatment Dropout | Delete | — |
| 33 | Chlamydia Antibiotic Resistance | Retain | Dependent Variable |
| 34 | Population Mobility | Retain | Autonomous Variable |
| 35 | Sexual Activity Level | Retain | Autonomous Variable |
| 36 | Health Education Coverage | Retain* | Relay Variable |
| 37 | Condom Use | Condom Promotion | Dependent Variable |
| **No.** | **Literature Variable** | **Interview Refining** | **MICMAC Quadrant** |
| 38 | Health Literacy | Awareness of Chlamydia Prevention and Control | Influential Variable |
| 39 | Willingness to Use Health Services | Delete | — |
| 40 | Sexual Stigma | Stigma | Autonomous Variable |
| 41 | Socioeconomic and Healthcare Level | Delete | — |
| 42 | Policy Requirements | Retain* | Influential Variable |
| 43 | Government Investment Amount | Retain | Relay Variable |
| 44 | Public Health Service Coverage | Delete | — |
| 45 | Free Public Health Services | Delete | — |
| 46 | Public Health Service Capacity | Delete | — |
| 47 | Community Mobilization | Retain | Influential Variable |

*Indicates that it is a theme word in the qualitative analysis
